# Melancholic and reactive depression: a reappraisal of old categories

**DOI:** 10.1186/1471-244X-13-311

**Published:** 2013-11-16

**Authors:** Jin Mizushima, Hitoshi Sakurai, Yuya Mizuno, Masaki Shinfuku, Hideaki Tani, Kadunari Yoshida, Chisa Ozawa, Asako Serizawa, Natsuko Kodashiro, Shinya Koide, Atsumi Minamisawa, Eisaku Mutsumoto, Nobuhiro Nagai, Sachiko Noda, Genichiro Tachino, Tatsuichiro Takahashi, Hiroyoshi Takeuchi, Toshiaki Kikuchi, Hiroyuki Uchida, Koichiro Watanabe, Hiroki Kocha, Masaru Mimura

**Affiliations:** 1 Department of Neuropsychiatry, Keio University School of Medicine, 35 Shinanomachi, Shinjuku-ku, Tokyo 160-8582, Japan; 2 Zama Mental Clinic, 5-1684-3 Iriya, Zama-shi, Kanagawa 252-0024, Japan

**Keywords:** Antidepressant, Diagnosis, Melancholic depression, Newcastle scale, Reactive depression

## Abstract

**Background:**

The dominant diagnostic model of the classification of depression today is unitarian; however, since Kurt Schneider (1920) introduced the concept of endogenous depression and reactive depression, the binary model has still often been used on a clinical basis. Notwithstanding this, to our knowledge, there have been no collective data on how psychiatrists differentiate these two conditions. We therefore conducted a survey to examine how psychiatrists in Japan differentiate patients with major depressive disorder who present mainly with melancholic features and those with reactive features.

**Methods:**

Three case scenarios of melancholic and reactive depression, and one-in-between were prepared. These cases were designed to present with at least 5 symptoms listed in the DSM-IV-TR with severity being mild. We have sent the questionnaires regarding treatment options and diagnosis for those three cases on a 7-point Likert scale (1 = “not appropriate”, 4 = “cannot tell”, and 7 = “appropriate”). Five hundred and two psychiatrists from over one hundred hospitals and community clinics throughout Japan have participated in this survey.

**Results:**

The melancholic case resulted significantly higher than the reactive case on either antidepressants (mean ± SD: 5.9 ± 1.2 vs. 3.6 ± 1.7, p < 0.001), hypnotics (mean ± SD: 5.5 ± 1.1 vs. 5.0 ± 1.3, p < 0.001), and electroconvulsive therapy (mean ± SD: 1.5 ± 0.9 vs. 1.2 ± 0.6, p < 0.001). On the other hand, the reactive case resulted in significantly higher scores compared to the melancholic case and the one- in-between cases in regards to psychotherapy (mean ± SD: 4.9 ± 1.4 vs. 4.3 ± 1.4 vs. 4.7 ± 1.5, p < 0.001, respectively). Scores for informing patients that they suffered from “depression” were significantly higher in the melancholic case, compared to the reactive case (mean ± SD: 4.7 ± 1.7 vs. 2.2 ± 1.4, p < 0.001).

**Conclusions:**

Japanese psychiatrists distinguish between major depressive disorder with melancholic and reactive features, and thus choose different treatment strategies regarding pharmacological treatment and psychotherapy.

## Background

While the classification of depression has long been a contentious issue, the dominant diagnostic model today is unitarian (i.e. there is only one type of depression, which varies by severity) [1]. Both of the widely accepted diagnostic systems, the Diagnostic and Statistical Manual of Mental Disorders, Fourth Edition (DSM-IV) and the International Statistical Classification of Diseases and Related Health Problems, 10th Revision (ICD-10), are based on using symptom check list criteria for diagnosis and assessment of severity [2-4]. Similarly, several well-known clinical guidelines for major depressive disorder (MDD) recommend treatments solely depending on the severity of depression, but they do not refer to the presence of psychogenic depression [5,6].

Heterogeneity of patients with depression based on the criteria of the present DSM and ICD classification systems has been considered to be an impediment to effective clinical care, evaluation of new interventions, and research on pathophysiology [2]. Even recently, Ghaemi and Vohringer pointed out that most depressive conditions can be shown to be about equally genetic and environmental. They also state that neurotic depression that is synonymous to reactive depression has a completely different psychopathological picture from melancholia [7]. Melancholia is characterized by a number of features such as disturbances in affect, psychomotor disturbances, cognitive impairment, vegetative dysfunctions, and psychosis. It has also been named in a variety of ways, including “endogenous” depression [8]; in fact, in Japan, “melancholia” is considered synonymous with “endogenous”. Furthermore, they may also differ markedly in treatment response; melancholia is more likely responsive to at least some antidepressants compared to neurotic depression [7]. Moreover, the presence of melancholia has been reported to predict a poor response to psychotherapy and placebo and a relatively good response to antidepressants and electroconvulsive therapy [9].

Since Kurt Schneider introduced the concept of melancholic depression and reactive depression [10], the binary model classifying depression into two principal types has often been used on a clinical basis. In the 1960s, members of the so-called Newcastle school supported the binary view with their multivariate analyses and formed criteria, which divided depression into melancholic depression and reactive depression [11-13]. Even recently, these components are still expected to be critically important and useful in actual clinical settings as mentioned above. However, to our knowledge, there have been no collective data on how psychiatrists differentiate the diagnosis and treatment between melancholic depression and reactive depression. This information would be expected to improve our understanding of actual clinical practice for these psychiatric conditions and help us enhance our treatment strategies. Therefore, we set up an investigation to find out how psychiatrists in Japan differentiate clinical care between patients with MDD who present mainly with melancholic features and those with reactive features.

## Methods

This survey was conducted from November 1, 2010 until January 30, 2011. Psychiatrists working in psychiatric hospitals, general hospitals, university hospitals, and community clinics in Japan were asked to participate in this survey. Three imaginary case scenarios of melancholic depression, reactive depression, and one-in-between were prepared, and reviewed by expert psychiatrists (see Additional file 1) for the questionnaire. All three cases fulfill the diagnosis of MDD according to the DSM-IV-TR [3]; when making the cases, these cases were designed to present with at least 5 symptoms listed in the DSM-IV-TR with severity being mild. On the other hand, what made these three case scenarios different were the scores of the Newcastle Scale 2nd edition (NCS-II) (see Additional file 2) [12,13]. The NCS-II comprises two positively and seven negatively weighted items which are summed to give a diagnostic score, -19 and above indicating reactive depression, and -20 and below endogenous depression [12,13]. Melancholic, reactive, and one-in-between cases were prepared with target NCS-II scores of -40, 15 and -20, respectively. Subsequently, 11 trained psychiatrists independently rated these three cases; the numbers of depressive symptoms listed in the DSM-IV-TR in the melancholic, reactive, and one-in-between cases were fairly consistent among the raters with 5.1 ± 0.3, 5.1 ± 0.3, and 4.9 ± 0.3 (mean ± SD), respectively (detailed data available upon request). Similarly, the NCS scores of the melancholic, reactive, and one-in-between cases were also similar among the 11 raters with -39.1 ± 2.0, 14.0 ± 2.6, -17.1 ± 3.3 (mean ± SD), respectively. The inter-rater reliability of the NCS-II scores were very high among the 11 raters with a Cronbach's alpha of 0.999. In addition to case scenarios, we developed a 13-question survey regarding treatment options. In this questionnaire, the details of this study were first described. If the psychiatrists agreed to participate in this study and provided written informed consent, they were subsequently asked to read three case scenarios and respond to questions regarding treatment for those three cases on a 7-point Likert scale (1=”not appropriate”, 4=”cannot tell”, and 7=”appropriate”) (see Additional file 3). The survey took approximately an hour to complete. All questionnaires were sent and collected by mail or by hand, under an anonymous state. The following information was also collected: age, sex, years in practice, work locations, and possession of license of designated psychiatrist by the Ministry of Welfare and Labor. All participants provided written informed consent after complete description of the study while the primary objective of this study was not disclosed since it could have affected their response.

### Statistical analysis

The data was analyzed using the SPSS 21.0 (IBM, New York). Values of interest were compared among melancholic, reactive, and one-in-between cases by the Kruskal-Wallis test or Wilcoxon signed rank test when appropriate. In addition, values of interest within cases were also compared by the Kruskal-Wallis test or Wilcoxon signed rank test when appropriate. A multiple regression analysis was used to examine effects of psychiatrists' sex, years in practice, work locations (i.e. psychiatric hospitals, general hospitals, and community clinics), and possession of license of designated psychiatrists on all the questions for each case (i.e. melancholic, reactive, and one-in-between). A value of p < 0.05 (two-tailed) was considered statistically significant. Bonferroni’s multiple comparison was applied when appropriate. This study was approved by the Institutional Review Board of the Zama Mental Clinic, and all subjects provided informed consent after a complete description of the study. This study complied with the Code of Ethics of the World Medical Association, Declaration of Helsinki.

## Results

### Characteristics of participants

A total of 650 psychiatrists working in 108 different institutes (see Additional file 4) were asked to participate in this survey; of these, 502 psychiatrists agreed and completed the questionnaire (response rate: 77.2%). Two hundred and sixty-eight respondents (53.3%) were affiliated with psychiatric hospitals, 168 (33.4%) with general hospitals, 42 (8.4%) with community clinics, and 11 (2.2%) with other institutes such as government offices while affiliations were not mentioned in 13 replies (2.6%). Out of the respondents, the proportion of male psychiatrists was higher compared to female doctors (69.3% vs. 29.4%). The mean ± SD age and years in practice were 37.9 ± 12.8 years old and 14.5 ± 12.5 years, respectively. The possession of a license of designated psychiatrist by the Ministry of Welfare and Labor was reported in 321 (63.9%) respondents.

### Pharmacological treatment, electroconvulsive therapy, and psychotherapy

Details of the results are shown in Table 1. The use of antidepressants was positively endorsed by respondents for the melancholic and one- in-between cases, while they were more reluctant to use antidepressants for the reactive case. When scores for “antidepressants” and “anxiolytics” were compared within each case, psychiatrists significantly preferred antidepressants over anxiolytics in the melancholic case (p < 0.001). In contrast, in the reactive case, anxiolytics were more favored than antidepressants. The mean scores of hypnotics, electroconvulsive therapy (ECT), and antipsychotics in the melancholic case were significantly higher than those of the reactive case, respectively. Although the magnitude of the difference was minor, psychotherapy was given a significantly higher score, compared to that for the melancholic and one- in-between cases. When attitudes towards antidepressant and psychotherapy were compared within each case, antidepressants were considered more appropriate than psychotherapy in the melancholic case. In contrast, in the reactive case, psychotherapy was preferred over antidepressants.

**Table 1:** Treatment preference of 502 psychiatrists towards three cases

	**Mean ± SD**			**Kruskal-**	**Post-hoc, Mann–Whitney U, Bonferroni-corrected P < 0.017 (i.e. 0.05/3)**
	**Case1 endogenous**	**Case2**	**Case 3**	**Wallis,**	
		**One in between**	**Reactive**	**P value**	
Pharmacological treatment					
Antidepressant	5.9 ± 1.2	5.2 ± 1.4	3.6 ± 1.7	<0.001	Case1-Case2, Case1-Case3, Case2-Case3
Anxiolytic	4.2 ± 1.4	4.2 ± 1.24	4.4 ± 1.5	0.010	Case1-Case3, Case2-Case3
Hypnotic	5.5 ± 1.1	5.4 ± 1.1	5.0 ± 1.3	<0.001	Case1-Case3, Case2-Case3
Antipsychotics	2.0 ± 1.2	2.0 ± 1.2	1.7 ± 1.1	<0.001	Case1-Case3, Case2-Case3
Electroconvulsive therapy	1.5 ± 0.9	1.3 ± 0.7	1.2 ± 0.6	<0.001	Case1-Case3, Case2-Case3
Psychotherapy	4.3 ± 1.4	4.7 ± 1.5	4.9 ± 1.4	<0.001	Case1-Case2, Case1-Case3
Environmental adjustment					
Family intervention	4.6 ± 1.2	4.3 ± 1.6	3.5 ± 1.5	<0.001	Case1-Case2, Case1-Case3, Case2-Case3
Reduce his/her duties	5.6 ± 1.2	5.8 ± 1.1	3.5 ± 1.5	<0.001	Case1-Case2, Case1-Case3, Case2-Case3
Resting	5.7 ± 1.2	5.5 ± 1.3	3.6 ± 1.6	<0.001	Case1-Case2, Case1-Case3, Case2-Case3
Hospitalization	3.0 ± 1.3	2.7 ± 1.5	1.7 ± 1.0	<0.001	Case1-Case2, Case1-Case3, Case2-Case3
Diagnosis					
Inform as “depression”	4.7 ± 1.7	4.1 ± 1.7	2.2 ± 1.4	<0.001	Case1-Case2, Case1-Case3, Case2-Case3
Inform as “depressive state”	5.7 ± 1.1	5.4 ± 1.3	4.7 ± 1.6	<0.001	Case1-Case2, Case1-Case3, Case2-Case3
Inform as “not a disease”	1.7 ± 1.0	2.0 ± 1.2	3.7 ± 1.7	<0.001	Case1-Case2, Case1-Case3, Case2-Case3

1 = “not appropriate”, 4 = “cannot tell”, and 7 = “appropriate”.

### Environmental adjustment

Categories of environmental adjustment (i.e. family intervention, burden reduction, hospital admission, and resting) were consistently judged appropriate for the melancholic case compared to the reactive case; the differences in the scores were especially large for restricting workload and suggesting a leave from work (Table 1). Interestingly, the score for reducing workload in the one-in-between case was the highest among the three cases.

### Diagnosis

For the three ways of informing diagnoses, telling patients that they were in a “depressive state” marked the highest scores for melancholic, reactive, and one-in-between cases, respectively (Table 1). Scores for informing patients that they suffered from “depression” were significantly higher in the melancholic case compared to the reactive case. In comparison, for informing patients that their symptoms were “not a disease”, the mean score in the melancholic case was significantly lower than that in the reactive case.

### Effects physicians’ characteristics on their responses

According to the multiple regression analysis, years in practice and sex were found to have effects on some of their responses as follows: years in practice (with increasing years) on use of antidepressants for melancholic (β: 0.034, 95% CI: 0.014 - 0.053, p = 0.001), one-in-between (β: 0.033, 95% CI: 0.014 - 0.052, p = 0.001), and reactive (β: 0.051, 95% CI: 0.034 - 0.068, p < 0.001) and sex (female compared with male) on use of hypnotics for one-in-between (β: 0.462, 95% CI: 0.231 - 0.694, p < 0.001) and reactive (β: 0.428, 95% CI: 0.167 - 0.688, p = 0.001), and psychotherapy for reactive (β: 0.491, 95% CI: 0.217 - 0.765, p < 0.001). Their sex (female compared with male) showed effects on their responses on resting for one-in-between (β: 0.446, 95% CI: 0.187 - 0.704, p = 0.001). Moreover, their years in practice also showed effects on their responses in diagnoses: years in practice (with increasing years) on “not a disease” for melancholic (β: -0.015, 95% CI: -0.024 - -0.006, p = 0.001), “depressive disorder” for reactive (β: 0.033, 95% CI: 0.018 - 0.049, p < 0.001), and “not a disease” for reactive (β: -0.042, 95% CI: -0.058 - -0.026, p < 0.001).

## Discussion

This study sheds light on the overall attitude that Japanese psychiatrists have towards treating MDD with different clinical features. Historically, the term “melancholia” is recognized as a distinctive clinical syndrome with a defined underlying biology that is distinguishable from other mood disorders. Efforts to define melancholic, however, have declined in recent years following failures to validate a distinction between melancholia and non-melancholia [2]. In reality, MDD encompasses multiple subgroups that differ meaningfully in phenomenology, natural history, treatment response, and pathophysiology [2]. In this study, we hypothesized that even though our three cases shared common symptoms of MDD according to the DSM-IV, selected treatment would vary according to melancholic and reactive features in each case. Indeed, our results, similarly, indicate that 85% of Japanese psychiatrists deem antidepressants as appropriate for MDD patients with melancholic features, compared to only 40% in MDD patients with reactive features. Melancholia, which has notwithstanding the limitations discussed below, these results show that Japanese psychiatrists may distinguish treatment strategies when approaching MDD with melancholic and reactive features.

Interestingly, our results are in contrast with recommendations that are stated in current treatment guidelines for MDD. Most of the currently available treatment guidelines recommend a choice of treatment options based on the severity of the illness [14,15], and little attention is drawn on the classical sub-categories of depression within treatment guidelines (e.g., melancholic and reactive depression). Under the present diagnostic criteria, even though patients may be diagnosed as MDD of matching severity, these same patients may present with heterogeneous groups of symptoms. There have been some reports that support the notion that melancholic depression is often identified with severe depression, an assumption coming from the homogenous or unitarian position [16]. However, the imaginary cases presented in the present study were designed to be mild in severity according to the DSM-IV. On the other hand, the heterogeneous position suggests that some symptoms, such as psychomotor disturbances, indicate that subtypes are biologically distinct and not a function of severity [8]. In fact, depression can exist as a disease, a disorder, a syndrome, and/or be normal, or abnormal reaction to salient stressors. The findings that Japanese psychiatrists take different treatment approaches towards the three imaginary cases in this study unlikely fit with a single dimensional or single categorical model. Recent reports suggest that effectiveness of treatment should be assessed according to the primary nosological entity of the patients in question [17]. The results from the present study suggest that Japanese psychiatrists approach MDD patients with melancholic and reactive features as patients with different nosological entities, and thus may choose different treatment strategies from their clinical experience. This may not be unique to Japanese psychiatrists, considering that the recent American Psychiatric Association (APA) guideline for treatment of MDD recommends that factors such as psychosocial stressors and problems in interpersonal relations be considered when choosing treatments [18].

Although the dominant model for MDD today is unitarian, which states that different presentations of depression are all from the same spectrum and differ only in severity, psychiatrists have traditionally utilized the binary approach in the treatment of MDD. Our results may indicate that this is still the case for a majority of psychiatrists, at least in Japan. The debate of the idea of two depressions (e.g., melancholic and reactive) was very active almost four decades ago, and the epidemiological studies in the 1960s and 1970s found that the divisions of melancholic and neurotic depressions did not imply differences in outcomes. As a result, it had faded after the introduction of the DSM-III [19] , which followed this debate. It did not, however, completely disappeared, rather,it still seems to be present with psychiatrists in Japan. A recent report that comes from outside of Japan also supports this binary approach; Fink et al. considered the proposition that 'melancholia’, a time-tested diagnostic concept, should be reinstated as a defined mood disorder in psychiatric classification [2]. Other mood disorders would best be labeled 'Non-melancholic Mood Disorders’. Furthermore, they even emphasized that the term 'major depressive disorder’ should be discarded. The idea of choosing different treatment strategies for different subgroups of depression may not to be limited to Japan, and the pros and cons for this type of treatment warrant further investigations. In the present survey, anxiolytics were more favored than antidepressants for the reactive case. Typically, neurotic or reactive depression is associated with anxiety and mood reactivity, and highly sensitive to psychosocial stressors [7], It would also not be surprising if neurotic and major depressive disorders also differed in treatment response. In fact, Imlah conducted a four-week randomized controlled trial to compare the effectiveness of alprazolam to amitriptyline in patients with neurotic depression and found that alprazolam was superior to amitriptyline [20]. Moreover, comparison of side effects showed a significant difference in favor of alprazolam over amitriptyline [20]. However, easy use of benzodiazepines should be refrained from in light of their potentially serious side effects such as falls [21], cognitive impairment [22,23], and dependence [24,25]. Furthermore, recent data on such direct comparison between benzodiazepines and antidepressants, especially selective serotonin reuptake inhibitors (SSRIs), are not available in the literature. Still, these findings, combined together with our results, may suggest the need of revisiting potential merits and demerits of the use of benzodiazepines for patients with reactive depression.

The result of our study must be interpreted in light of a number of limitations. First, a possibility of selection bias cannot entirely be rejected. The respondents were distributed over a wide area of Japan, and the response rate was as high as approximately 80%, yet the survey is limited to psychiatrists who belong to a portion of institutes within our country. However, since our samples were collected from multiple areas all over Japan, our results are expected to be generally representative of Japanese psychiatrists’ practice. Second, the cases in this study are imaginary cases, and not actual cases. In real clinical settings, information on patients such as social history, family history, and relevant stressors can be collected more thoroughly. Furthermore, demographic and clinical features such as age, sex, duration of illness, and presentation of our case scenarios may have affected the clinical treatment choice; for example, this might have happened because only the second case mentioned overwork, and in the other two other cases, no mention was made of it. In addition, the neurotic case may be little obvious; as a result, responders may have been led, to some extent, to choose psychotherapy and prescribe some anxiolytics to this young, forlorn man who has no history of previous psychiatric illness. Moreover, a more thorough longitudinal assessment of such a patient would have been ideal. Third, we showed three cases in succession, so respondents had time to compare the clinical features of the three cases intentionally, which may have affected their responses. Fourth, it would have been ideal to ask reasons for their responses in the present study although the response rate may have been compromised. Finally, physician's treatment decisions are expected to be subject to their educational and training background as well as direct and indirect influence of current and local standard of care, which indicates the need of further investigations in order to provide a robust agreement on this issue.

## Conclusions

The findings from this study indicate that Japanese psychiatrists distinguish between MDD with melancholic and reactive features, and thus choose different treatment strategies regarding psychopharmacology and psychotherapy. Thorough understanding of the differences in psychiatrists’ practice between melancholic and reactive depressions that we observed in this study is critically important to appraise the current treatment strategies for MDD. In light of a paucity of the data on this clinically relevant issue, merits and demerits of these different treatment strategies require further investigations in order to devise optimal treatment for MDD that present with different clinical features.

## Competing interests

Dr. Uchida has received grants from Pfizer, Astellas Pharmaceutical, Eisai, Otsuka Pharmaceutical, GlaxoSmithKline, Shionogi, and Dainippon-Sumitomo Pharma, Eli Lilly, Mochida Pharmaceutical, Meiji-Seika Pharma, Janssen Pharmaceutical, and Yoshitomi Yakuhin and speaker’s honoraria from Otsuka Pharmaceutical, Janssen Pharmaceutical, Novartis Pharma, Eli Lilly, Shionogi, GlaxoSmithKline, Yoshitomi Yakuhin, Dainippon-Sumitomo Pharma, and Janssen Pharmaceutical within the past two years. Dr. Watanabe has received grants, or consultant fees from Dainippon Sumitomo Pharma, Eli Lilly, Otsuka Pharmaceutical, and Mochida GlaxoSmithKline, Mitsubishi Tanabe Pharma, Meiji Seika Pharma, and Pfizer, and received speaker’s honoraria from Eli Lilly, GlaxoSmithKline, Janssen Pharmaceutical, Meiji, Otsuka Pharmaceutical, Pfizer, Shionogi, and Mochida within the past three years. Dr. Mimura has received grants, or consultant fees from Abbott and Pfeizer and received speaker’s honoraria from Asahi Kasei, Astellas Pharma, Chemiphar, Daiichi Sankyo, Eisai, Eli Lilly, GlaxoSmithKline, Janssen Pharmaceutical, Meiji, Mochida, MSD, Novartis, Otsuka Pharmaceutical, Shionogi, Tsumura, Yoshitomi Yakuhin within the past three years. Other authors have nothing to disclose.

## Authors’ contributions

JM, HS, and YM conceived and designed the study. KW and HU provided supervision. NK, SK, AM, EM, NN, SN, GT, TT analyzed and interpreted the data. MS, KY, and HU checked for statistical inconsistency, and interpreted the results. JM, HS, YM, MS, HT, and CT drafted the report. HT, TK, HU, HK, and MM critically reviewed the report. All authors read and approved the final version of the report. The corresponding author had full access to all the data in the study, and had final responsibility for the decision to submit for publication.

## Pre-publication history

The pre-publication history for this paper can be accessed here:

http://www.biomedcentral.com/1471-244X/13/311/prepub

## Supplementary Material

Additional file 1 Three case scenarios: melancholic depression, reactive depression, and one-in-between. Click here for file

Additional file 2 Table for the Newcastle Scale 2nd edition (NCS-II). Click here for file

Additional file 3 Questionnaire sent to the psychiatrists. Click here for file

Additional file 4 List of participating centers in this study. Click here for file
