# Supplementary material for: Melancholic and reactive depression: a reappraisal of old categories
Source: BMC Psychiatry. 2013 Nov 16;13:311. doi: 10.1186/1471-244X-13-311 (PMC3840623; doi:10.1186/1471-244X-13-311)
Supplement: Additional file 1 — Three case scenarios: melancholic depression, reactive depression, and one-in-between. [file 1471-244X-13-311-S1.docx]

Additional file 1: Three case scenarios: melancholic depression, reactive depression, and one-in-between

**Case 1:**

Melancholic depression

Number of the DSM-IV-defined depressive symptoms in this case: 5.1±0.3 (mean±SD)

NCS-II score: -39.1±2.0 (mean±SD)

A 25 year–old business man

No past history of physical or psychiatric illness.

He has experienced a depressive mood for the last two weeks. He cannot recognize any causes. Although he feels that he needs more sleep, he wakes up early in the morning. He has lost his appetite, and as a result, he has lost 2 kilograms in weight in these two weeks. He always feels tired, and he cannot recover by resting. He often went to the movies before, but nowadays, he is not willing to do so. He no longer watches baseball games on TV, which he always did. His depressive mood stays nearly all day, but it is especially severe in the morning. He handles his jobs even though he finds it tough. Since he often sighs, one of his friends encouraged him to visit a psychiatrist, which he did today. He has never thought about committing suicide, and he has never experienced any manic episodes.

**Case 2:**

In-between case of melancholic depression and reactive depression

Number of the DSM-IV-defined depressive symptoms in this case: 4.9±0.3 (mean±SD)

NCS-II score: -17.1±3.3 (mean±SD)

A 25 year–old business man

No past history of physical or psychiatric illness.

He has experienced a depressive mood for the last two weeks. He had a job transfer a month ago. Although the new work is longer in time and more competitive, he does not feel stressed because the new work was wished for and granted. Although he feels that he needs more sleep, he wakes up early in the morning. He has lost his appetite, and as a result, he has lost 2 kilograms in weight in these two weeks. He always feels tired, and he cannot recover by resting. He often went to the movies before, but nowadays, he is not willing to do so. He no longer watches baseball games on TV, which he always did. His depressive mood stays nearly all day. He handles his jobs even though he finds it tough. Since he often sighs, one of his friends encouraged him to visit a psychiatrist, which he did today. He has never thought about committing suicide, and he has never experienced any manic episodes.

**Case 3:**

Reactive depression

Number of the DSM-IV-defined depressive symptoms in this case: 5.1±0.3 (mean±SD)

NCS-II score: 14.0±2.6 (mean±SD)

A 25 year–old business man

No past history of physical or psychiatric illness.

He has experienced a depressive mood for the last two weeks after his girlfriend left him. He feels he cannot sleep deeply, and he wakes up several times a night. He cannot get up in the morning. He has lost his appetite, and as a result, he has lost 2 kilograms in weight in these two weeks. He always feels tired, and he cannot recover by resting. He often went to the movies before, but nowadays, he is not willing to do so because memories of his ex-girlfriend come to mind. He no longer watches baseball games on TV, which he always did. He always thinks of her, and time flies. He handles his jobs even though he finds it tough. He can enjoy going out for drinks with his friends, but when he comes home – which is dark and lonely – he feels even more depressed. He became worried about this depressive mood, and he decided to visit a psychiatrist today. He has never thought about committing suicide, and he has never experienced any manic episodes.
