# Supplementary material for: Melancholic and reactive depression: a reappraisal of old categories
Source: BMC Psychiatry. 2013 Nov 16;13:311. doi: 10.1186/1471-244X-13-311 (PMC3840623; doi:10.1186/1471-244X-13-311)
Supplement: Additional file 2 — Table for the Newcastle Scale 2nd edition (NCS-II). [file 1471-244X-13-311-S2.docx]

Additional file 2: Table for the Newcastle Scale 2nd edition (NCS-II)

Items Grade Score

_______________________________________________________

Sudden onset Yes -6

No 0

Duration Under 3 months -6

3 months – under 1 year -4

1 year – under 2 years -2

2 years and over 0

Psychological stress Severe +12

Mild/moderate + 6

None 0

Situational phobias Marked/incapacitating + 8

Mild/moderate + 4

None 0

Persistent depression Yes - 2

None 0

Depression worse A.M. Yes - 2

No 0

Early waking Yes -10

No 0

Retardation Yes - 9

No 0

Delusions Yes -7

No 0

__________________________________________________________

Total score ranges:

-19 and above = reactive depression

-20 and below = endogenous (or melancholic) depression
