# Supplementary material for: Melancholic and reactive depression: a reappraisal of old categories
Source: BMC Psychiatry. 2013 Nov 16;13:311. doi: 10.1186/1471-244X-13-311 (PMC3840623; doi:10.1186/1471-244X-13-311)
Supplement: Additional file 3 — Questionnaire sent to the psychiatrists. [file 1471-244X-13-311-S3.doc]

Additional file 3: Questionnaire sent to the psychiatrists

Question: After reviewing each case scenario, please rate the appropriateness of each treatment and diagnosis in the score scale of 7. (1=”not appropriate”, 4=”cannot tell”, and 7=”appropriate”).

Not appropriate　 　　Cannot　tell　　　Appropriate

1 2 3 4 5 6 7

<Treatment>

1. Prescribe antidepressants

2. Prescribe anxiolytics

3. Prescribe hypnotics

4. Prescribe antipsychotics

5. Perform electroconvulsive therapy

6. Perform psychotherapy

<Environmental Adjustment>

1. Family intervention

2. Restrict workload

3. Suggest a leave from work

4. Hospitalization

<Diagnosis>

1. Inform as “depression”

2. Inform as “depressive state”

3. Inform as “not a disease”
