# Supplementary material for: Melancholic and reactive depression: a reappraisal of old categories
Source: BMC Psychiatry. 2013 Nov 16;13:311. doi: 10.1186/1471-244X-13-311 (PMC3840623; doi:10.1186/1471-244X-13-311)
Supplement: Additional file 4 — List of participating centers in this study. [file 1471-244X-13-311-S4.docx]

Additional file 4: List of participating centers in this study

(1) Psychiatric hospitals and community psychiatric clinics

Akabane Tanaka Clinic (Tokyo, Japan), Akasaka Mental Clinic (Tokyo, Japan), Akebono Clinic (Tokyo, Japan), Amekudai Hospital (Okinawa, Japan), Aoyama Shibuya Mental Clinic (Tokyo, Japan), Arakaki Hopital (Okinawa, Japan), Arakawa Mental Clinic (Tokyo, Japan), Asaka Hospital (Fukushima, Japan), Ashikari Clinic (Tokyo, Japan), Chigasaki Clinic (Kanagawa, Japan), Denenchofu Clinic (Tokyo, Japan), Ebisu Icchoume Clinic (Tokyo, Japan), Feel Fine Clinic (Kanagawa, Japan),

Fujii Shinryoujo (Tokyo, Japan), Gaia Crystal Clinic (Kanagawa, Japan), Ginza Mental Clinic (Tokyo, Japan), Ginza Taimei Clinic (Tokyo, Japan), Hachiouji Keiai Hospital (Tokyo, Japan), Higashi Ginza Clinic (Tokyo, Japan), Hizen Psychiatrtic Center (Saga, Japan), Hozumi Clinic (Tokyo, Japan), Iidabashi Garden Clinic (Tokyo, Japan), Ikegami Clinic (Tokyo, Japan), Inokashira Hospital (Tokyo, Japan), Isao Clinic (Saitama, Japan), Isono Iin (Ibaraki, Japan), Iwata Clinic (Tokyo, Japan),

Jikei Hospital (Okayama, Japan), Jiundo-Naika Hospital (Tokyo, Japan), Jiyugaoka Mental Clinic (Tokyo, Japan), Jiyugaoka Takagi Clinic (Tokyo, Japan), Kamatori Mental Clinic (Chiba, Japan), Kamei Clinic (Tokyo, Japan), Kamisuwa Hospital (Nagano, Japan), Kawachi Clinic (Tokyo, Japan), Kawasaki City Psychiatric Rehabilitation Center (Kanagawa, Japan), Kei Clinic (Tokyo, Japan), Keiai Clinic (Tochigi, Japan), Kimura Clinic (Shizuoka, Japan), Kinosaki Hospital (Chiba, Japan),

Komagino Hospital (Tokyo, Japan), Komazawa Kei Clinic (Tokyo, Japan), Komoro Kogen Hospital (Nagano, Japan), Kounuma Clinic (Saitama, Japan), Kunugi Clinic (Yamanashi, Japan), Kurachi Clinic (Kanagawa, Japan), Kusatsu Hospital (Tochigi, Japan), Makino Clinic (Tokyo, Japan), Matsumi Hospital (Tokyo, Japan), Mental Health Tai Clinic (Saitama, Japan), Midorinokaze Mental Clinic (Tokyo, Japan),

Mitaka Ekimae Kokorono Clinic (Tokyo, Japan), Mitaka Mental Clinic (Tokyo, Japan), Miura Shinryoujo (Tokyo, Japan), Mizushima Hiroko Kokorono Kenko Clinic (Tokyo, Japan), Motoatsugi Masuda Clinic (Tokyo, Japan), Musashino Central Hospital (Tokyo, Japan), Musashino Hospital (Saitama, Japan), Nakajima Clinic (Tokyo, Japan), Nakayama Clinic (Tokyo, Japan), Nakayama Hospital (Chiba, Japan),

National Center for Global Health and Medicine Kohnodai Hospital (Chiba, Japan),

National Hospital Organization Chiba Medical Center (Chiba, Japan), National Hospital Organization Kurihama Medical and Addiction Center (Kanagawa, Japan),

Noguchi Clinic (Kanagawa, Japan), Odori Tsugeno Clinic (Hokkaido, Japan), Oizumi Hospital (Tokyo, Japan), Oizumi Mental Clinic (Tokyo, Japan), Omiyama Clinic (Tokyo, Japan), Ootemachi Mental Clinic (Tokyo, Japan), Roujyu Sanitarium (Osaka, Japan), Sagiya Mental Clinic (Saitama, Japan), Saito Hospital (Tokyo, Japan),

Sakuragaoka Kinen Hospital (Tokyo, Japan), Sanaruko Hospital (Shizuoka, Japan)

Sankei Hospital (Tokyo, Japan), Sawa Hospital (Osaka, Japan), Seijo Mental Clinic (Tokyo, Japan), Seijo Sumioka Clinic (Tokyo, Japan), Seiwa Hospital (Tokyo, Japan),

Senba Shinryoujo (Ibaraki, Japan), Senzoku Clinic (Tokyo, Japan), Senzoku Mental Clinic (Tokyo, Japan), Shakujii Kouen Clinic (Tokyo, Japan), Shibuya Moriya Clinic (Tokyo, Japan), Shimousa Pyschiatric Center (Chiba, Japan), Shinjuku Higashi Mental Clinic (Tokyo, Japan), Shinjuku Minami Shinryoujo (Tokyo, Japan), Shinyurigaoka Nakamura Clinic (Kanagawa, Japan), Shioiri Shinkeika Iin (Tokyo, Japan), Sho Midori Hospital (Saitama, Japan), Showa University Northern Yokohama Hospital Mental Care Center (Kanagawa, Japan), Sugamo Kimura Clinic (Tokyo, Japan), Susei Healing Center (Kanagawa, Japan), Suzuki Shinkeika Clinic (Kanagawa, Japan), Suzuki Shinkeika Shinryoujo (Tokyo, Japan), Tabata Jinsei Clinic (Tokyo, Japan), Takahashi Clinic (Tokyo, Japan), Takamiya Hospital (Miyazaki, Japan)

Takeda Hospital (Tokyo, Japan), Tashiro Clinic (Saitama, Japan), Tokorozawa Muashino Clinic (Saitama, Japan), Tokui Kinen Gotanda Mental Clinic (Tokyo, Japan), Tokyo Musashino Hospital (Tokyo, Japan), Tokyo-Kaido Hospital (Tokyo, Japan), Toranomon Ryu Clinic (Tokyo, Japan), Toyama Chuou Shinryoujo (Toyama, Japan), Toyoko Keiai Hospital (Kanagawa, Japan), Tsurugaoka Garden Clinic (Tokyo, Japan), Tsutsuji Mental Hospital (Gunma, Japan), Uchida Clinic (Tokyo, Japan), Warabi Mental Clinic (Saitama, Japan), Watanabe Iin (Kanagawa, Japan), Yaesu Mental Clinic (Tokyo, Japan), Yamada Hospital (Tokyo, Japan), Yamanashi Prefectural Kita Hospital (Yamanashi, Japan), Yanai Clinic (Tokyo, Japan)

Yokohama Maioka Hospital (Tokyo, Japan), Yokohama Okanoue Hospital (Kanagawa, Japan), Yokoyama Clinic (Tokyo, Japan), Yomiuri Land Keiyu Hospital (Tokyo, Japan), Yoyogi no Mori Shinryoujo (Tokyo, Japan), Yuwa Mental Clinic (Tokyo, Japan)

(2) General hospitals

Ashikaga Red Cross Hospital (Tochigi, Japan), Hino Municipal Hospital (Tokyo, Japan), Iida Hospital (Nagano, Japan), Kawakita General Hospital (Tokyo, Japan),

Kawasaki Municipal Ida Hospital (Kanagawa, Japan), Kawasaki Municipal Kawasaki Hospital (Kanagawa, Japan), Keiyu Hospital (Kanagawa, Japan), Kitasato Institute Hospital (Tokyo, Japan), National Center for Child Health and Development (Tokyo, Japan), National Hospital Organization Ryukyu Hospital (Okinawa, Japan), National Hospital Organization Tokyo Medical Center (Tokyo, Japan), Nippon Kokan Hospital (Kanagawa, Japan), Saitama Social Insurance Hospital (Saitama, Japan), Sakuramachi Hospital (Tokyo, Japan), Seibo Hopital (Tokyo, Japan), Shimada Ryoiku Center (Tokyo, Japan), Shizuoka Red Cross Hospital (Shizuoka, Japan), Tachikawa Hospital (Tokyo, Japan), Tokyo Kosei Nenkin Hospital (Tokyo, Japan), Tokyo Metropolitan Ohtsuka Hospital (Tokyo, Japan), Yokohama Municipal Citizen’s Hospital (Kanagawa, Japan),

(3) University hospitals

Kagoshima University (Kagoshima, Japan), Keio University (Tokyo, Japan),

Kyoto Prefectural University (Kyoto, Japan), Kyushu University (Fukuoka, Japan), Nagoya University (Aichi, Japan), Okayama University (Okayama, Japan), Showa University (Tokyo, Japan), Toho University (Tokyo, Japan), Tokyo Dental College Ichikawa General Hospital (Chiba, Japan), Tokyo Women’s Medical University (Tokyo, Japan)
